# The Induction of Alpha-1 Antitrypsin by Vitamin D in Human T Cells Is TGF-β Dependent: A Proposed Anti-inflammatory Role in Airway Disease

**DOI:** 10.3389/fnut.2021.667203

**Published:** 2021-08-12

**Authors:** Yin-Huai Chen, Charlotte E. Cheadle, Louise V. Rice, Paul E. Pfeffer, Sarah Dimeloe, Atul Gupta, Andrew Bush, Bibek Gooptu, Catherine M. Hawrylowicz

**Affiliations:** ^1^Peter Gorer Department of Immunobiology (Formerly Asthma, Allergy and Lung Biology), School of Immunology and Microbial Sciences, King's College London, London, United Kingdom; ^2^Medical Research Council and Asthma UK Centre for Allergic Mechanisms of Asthma, Guy's Hospital, King's College London, London, United Kingdom; ^3^National Heart and Lung Institute, Royal Brompton & Harefield National Health Service Foundation Trust, London, United Kingdom; ^4^Centre for Paediatrics and Child Health, National Heart and Lung Institute, Imperial College, Royal Brompton Hospital, London, United Kingdom; ^5^National Institute for Health Research Leicester Biomedical Research Centre-Respiratory and Leicester Institute of Structural & Chemical Biology, University of Leicester, Leicester, United Kingdom; ^6^London Alpha-1 Antitrypsin Deficiency Service, Royal Free Hospital, London, United Kingdom

**Keywords:** vitamin D, T cell, alpha-1 antitrypsin (AAT), anti-inflammatory, HBEC, airway disease

## Abstract

**Background:** Vitamin D upregulates anti-inflammatory and antimicrobial pathways that promote respiratory health. Vitamin D synthesis is initiated following skin exposure to sunlight, however nutritional supplementation can be required to address deficiency, for example during the winter months or due to cultural constraints. We recently reported that 1α,25-dihydroxyvitamin D3 (1,25(OH)_2_D3) treatment induced alpha-1 antitrypsin (AAT) expression in CD4+, but not CD8+ T cells, with evidence supporting an immunoregulatory role.

**Research Question:** To understand the relationship between vitamin D, lung AAT levels and T lymphocytes further we investigated whether TGF-β is required as a co-factor for 1,25(OH)_2_D3-induced upregulation of AAT by vitamin D in CD8+ T cells *in vitro* and correlated circulating vitamin D levels with lung AAT levels *in vivo*.

**Results:** 1,25(OH)_2_D3 in combination with TGF-β1 increased AAT expression by CD8+ T cells, as well as *VDR* and *RXR*α gene expression, which may partly explain the requirement for TGF-β. CD4+ T cells may also require autocrine stimulation with TGF-β as a co-factor since 1,25(OH)_2_D3 was associated with increased TGF-β bioactivity and neutralisation of TGF-β partially abrogated 1,25(OH)_2_D3-induced *SERPINA1* gene expression. Neither CD4+ nor CD8+ T cells responded to the circulating vitamin D precursor, 25-hydroxyvitamin D3 for induction of *SERPINA1*, suggesting that local generation of 1,25(OH)_2_D3 is required. Transcriptional gene profiling studies previously demonstrated that human bronchial epithelial cells rapidly increased TGF-β2 gene expression in response to 1,25(OH)_2_D3. Here, human epithelial cells responded to precursor 25(OH)D3 to increase bioactive TGF-β synthesis. CD8+ T cells responded comparably to TGF-β1 and TGF-β2 to increase 1,25(OH)_2_D3-induced AAT. However, CD8+ T cells from adults with AAT-deficiency, homozygous for the Z allele of *SERPINA1*, were unable to mount this response. AAT levels in the airways of children with asthma and controls correlated with circulating 25(OH)D3.

**Conclusions:** Vitamin D increases AAT expression in human T cells and this response is impaired in T cells from individuals homozygous for the Z allele of *SERPINA1* in a clinic population. Furthermore, a correlation between circulating vitamin D and airway AAT is reported. We propose that vitamin D-induced AAT contributes to local immunomodulation and airway health effects previously attributed to vitamin D.

## Introduction

Vitamin D is increasingly recognised as an important factor in regulating immunity and respiratory health, and vitamin D deficiency is associated with several immune-mediated airway diseases, including asthma and chronic obstructive pulmonary disease (COPD) (1–3). This is attributed to its diverse immuno-modulatory functions including the capacity to promote anti-microbial pathways, suppress inflammatory responses while enhancing immuno-modulatory functions such as anti-inflammatory IL-10 production (4, 5).

Alpha-1 antitrypsin (AAT) plays a critical role in protecting lung parenchymal tissue from direct elastinolytic degradation and pro-inflammatory effects of serine proteases, notably neutrophil elastase (6). It is predominantly synthesized in the liver by hepatocytes and is secreted into the plasma as the most abundant circulating antiprotease. The metastable structure of native AAT and other members of the serine protease inhibitor (serpin) superfamily provides the potential for stabilizing conformational change (7). This is utilized in the functional mechanism of protease inhibition, but also renders AAT vulnerable to point mutations, as seen in the Glu342Lys (Z) variant. Within hepatocytes, that are the major source of systemic AAT, this triggers misfolding in the endoplasmic reticulum, aberrant conformational change and stabilizing intermolecular linkage to form AAT polymers (8). The results are a deficiency of circulating functional AAT, and retention of misfolded and polymerized AAT within hepatocytes associated with pro-inflammatory and pro-fibrotic gain-of-function (9). This combination predisposes Z variant homozygotes (denoted Pi^*^ZZ) to severe and early-onset COPD and liver disease (hepatitis, cirrhosis, and hepatocellular carcinoma) (10). The lung disease seems predominantly driven by loss-of-function mechanisms though extracellular polymers encourage neutrophil chemotaxis and activity and are found in the circulation and lung (11).

AAT displays multiple immuno-regulatory functions including inhibition of neutrophil chemotaxis (12), induction of IL-1Ra by macrophages (13) and the development of tolerogenic dendritic cells and their production of IL-10 (14, 15). We have recently shown the requirement of AAT in the 1,25(OH)_2_D3-mediated induction of IL-10 in CD4+ T cells (16). In addition to immunomodulatory effects mediated by antiprotease function, immunomodulatory effects of AAT that are independent of antiprotease function are now recognized (17). The absence of these effects may further exacerbate the pro-inflammatory state in lungs of individuals with AAT deficiency.

Cells within the lungs e.g., alveolar epithelial cells, monocytes and neutrophils are capable of secreting AAT to supplement liver-derived AAT in the airways (18–20). We have demonstrated that the active form of vitamin D, 1,25(OH)_2_D3, upregulates expression of *SERPINA1*, the gene encoding the serine protease inhibitor AAT, and AAT protein secretion by human primary CD4+ T cells (16). Conversely this was not seen for monocytes. Moreover, vitamin D reduces secretion of matrix metalloproteinase (MMP-)9 that degrades AAT (21, 22). These data suggest that vitamin D availability may boost AAT levels within the airway microenvironment and so represent an additional mechanism by which vitamin D protects the airways.

Our published data demonstrate that 1,25(OH)_2_D3 alone does not upregulate AAT in human CD8+ T cells (16). Given the proposed role of CD8+ T cells in chronic airway conditions such as COPD (23), we investigated whether a co-factor that acts in concert with 1,25(OH)_2_D3 may induce AAT synthesis by human CD8+ T cells. TGF-β was investigated, since previous studies reported the capacity of hepatoma cell lines and human lung epithelial cell lines to produce AAT in response to TGF-β (24, 25). Furthermore, TGF-β and vitamin D are reported to act co-operatively. Independent studies report, for example, that TGF-β increases gene and protein expression for VDR (26) and the enzyme CYP27B1, which converts precursor vitamin D, 25(OH)D, to the active moiety, 1,25(OH)_2_D3 (27, 28). Furthermore, 1,25(OH)_2_D3 in combination with TGF-β increases the frequency of CD4+ Foxp3+ Treg cells (29). These data provide the rationale for the present study to investigate whether 1,25(OH)_2_D3 and TGF-β interact with each other for enhanced effects in human CD8+ T cells.

## Materials and Methods

### Subject Characteristics

Healthy donors (“Human peripheral blood cell laboratory studies to investigate the role of immune and inflammatory pathways in respiratory disease” by the local research ethics committee, REC reference: 14/LO/1699) were recruited and provided full written informed consent with full Research Ethics Committee approval. COPD patients without AAT deficiency (Chest clinic, Guy's Hospital, 14/LO/1699), and PiZZ-AAT deficient patients with or without COPD (Targeting dysfunctional mechanisms in alpha-1 antitrypsin deficiency, The Royal Free Alpha-1 clinic, REC reference: 13/LO/1085) were recruited and provided full written informed consent with full Research Ethics Committee approval, including anonymization of donors to researchers. The study in a pediatric asthma cohort and control children undergoing diagnostic bronchoscopy for non-asthma related purposes was approved by the Royal Brompton and Harefield Research Ethics Committee (09/H07008/48) which has been previously described Gupta et al. (30). Informed consent was obtained from parents and age-appropriate assent from children. Serum levels of 25-hydroxyvitamin D were measured as previously described by Gupta et al. (30). All work adhered strictly to institutional safety guidelines and procedures. Table 1 provides data on the number of individuals studied throughout, and figure legends identify number of individual donors in each experimental series.

**Table 1:** Clinical parameters of healthy control, COPD, and PiZZ patients.

**CD4+ T cells**	**Healthy**	**COPD**	**PiZZ**	**CD8+ T cells**	**Healthy**	**COPD**	**PiZZ**
N number	6	9	6	N number	12	9	20
Gender (F:M)	4:2	5:4	4:2	Gender (F:M)	6:6	5:4	9:11
Age range	24–58	53–79	18–62	Age range	26–63	53–79	18–75
Lung function				Lung function			
FEV1%	Expected to be normal	28–94	27–120	FEV1%	Expected to be normal	28–94	27–125
FEV1/FVC %		27–69	39–81	FEV1/FVC %		27–69	20–83

### Cell Isolation and Culture

Human peripheral blood mononuclear cells (PBMCs) were isolated, as previously described Xystrakis et al. (31). CD4+ and CD8+ T cells were isolated using Dynabeads CD4 or CD8 positive selection kits (Invitrogen, Paisley, UK). Cells (1 × 10^6^/ml) were cultured in RPMI 1640 medium supplemented with 10% FCS, 2 mM L-glutamine, and 50 μg/ml gentamicin, and stimulated with plate-bound anti-CD3 (1 μg/ml; OKT3) plus 50 IU/ml recombinant human IL-2 (Eurocetus, UK) in the presence or absence of 1,25(OH)_2_D3 (BIOMOL research labs, UK), TGF**-**β1 (R&D Systems, UK), TGF**-**β2 (R&D Systems) or anti-TGF**-**β (R&D Systems) at indicated concentrations for 7 days. NIST SRM1648a Urban Particulate Matter (National Institute of Standards & Technology, USA) is an urban total particulate matter reference material with mean particle diameter 5.85 μm, collected in the USA. NIST was prepared as previously described by Pfeffer et al. (32).

### HBEC Isolation and Culture Conditions

Primary human bronchial epithelial cells (HBECs) were acquired and maintained as previously described Pfeffer et al. (33). HBECs were stimulated with 50 μg SRM1648a, a standard reference urban particulate matter, NIST, in the absence or presence of 10, 100, or 1,000 nM 25(OH)D3 for 24 h. Lysed cell monolayers were collected for assessment by qRT-PCR. HBECS were also stimulated with 25(OH)D3 (1 μM) for 48 h and culture supernatant collected for TGF-β bioassay.

### qRT-PCR

RNA was extracted from cell pellets using the Rneasy Mini kit (Qiagen, Crawley UK) according to the manufacturer's instructions. Nanodrop ND-1000 spectrophotometer was used to quantify RNA. 250 ng of RNA was reverse transcribed into cDNA. Quantitative real-time PCR was performed, as previously described Urry et al. (34) in triplicates by using an Applied Biosystems 7900 HT system and FAM-labeled Assay-on-Demand reagent sets for *SERPINA1* (Hs01097800_m1), *VDR* (Hs01045846_m1), *RXR*α (Hs01067640_m1). Quantitative real-time PCR reactions were multiplexed with VIC-labeled 18S primers and probes (Hs99999901_s1) as an endogenous control and analysed with SDS software, version 2.1 (Applied Biosystems, Foster City, Calif), according to 2^−ΔCt^ (× 10^6^).

### AAT ELISA

The AAT ELISA employed a commercial polyclonal rabbit anti-human AAT primary capture antibody (Dako, UK) and a biotin-conjugated rabbit anti-human AAT secondary detection antibody [in-house purified, previously described in Dimeloe et al. (16)]. Standards were serially diluted in RPMI medium plus 10% foetal calf serum, by serial 1:2 dilutions from the top standard of 200 ng/ml using human plasma-purified AAT (Sigma-Aldrich, UK). Streptavidin-alkaline phosphatase (Sigma-Aldrich, UK) and 4-Nitrophenyl phosphate disodium salt hexahydrate (1 mg/ml; Sigma-Aldrich, UK) were used to detect AAT. Absorbance was measured at 405 nm on an Anthos HTII plate reader (Anthos, UK) using Softmax pro software and quantified using GraphPad Prism version 6 (GraphPad software Inc., USA). The lower limit of detection was 0.32 ng/ml.

### TGF-β Bioassay

TGF-β bioactivity was measured using mink lung epithelial cell lines (MLECs) transfected with plasminogen activator inhibitor-1, a TGF-β target gene, fused with the firefly luciferase construct (kindly donated by Professor Daniel Rifkin, New York University). Briefly, 3 × 10^5^ MLECs/ml were incubated for 14 h at 37°C in 5% CO_2_ with cell culture supernatants or standards. Culture media was aspirated, washed, cells lysed and luciferase activity determined using Firefly Luciferase Assay Kit (Biotium, USA) and measured by luminescence (1450 MicroBeta TriLux; PerkinElmer, USA) in accordance with the manufacturer's instructions. Light emitted corresponds to the amount of bioactive TGF-β. Serum free RPMI was used for these experiments.

### Data Analysis

Data are shown as mean ± standard error of mean (SEM) unless otherwise indicated.

Data analysis was performed using Graphpad Prism version 6.00 for Mac OS X (Graphpad software Inc., USA). Statistical test used as in relevant figure legend.

## Results

### Exogenous TGF-β1 and 1,25(OH)_2_D3 Act Cooperatively to Enhance *SERPINA1*/AAT in CD8+ T Cells

**Figure 1:**
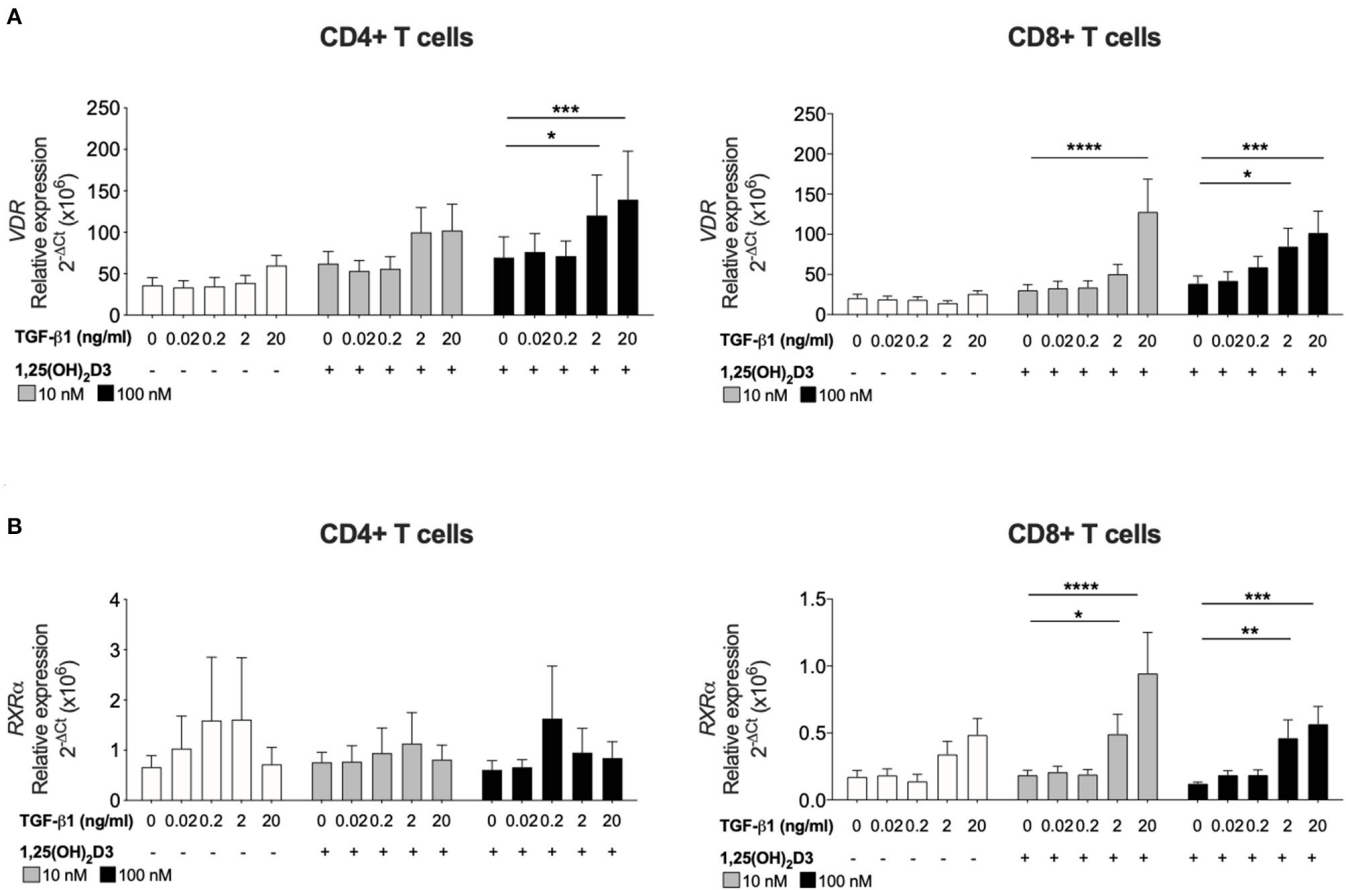
TGF-β1 together with 1,25(OH)_2_D3 upregulates *VDR* and *RXR*α gene expression by CD8+ T cells. CD4+ and CD8+ T cells (*n* = 6) were stimulated and cultured for 7 days in the absence or presence of 10 or 100 nM 1,25(OH)_2_D3, with or without TGF-β1 (0.02, 0.2, 2, 20 ng/ml). Gene expression of **(A)** *VDR* and **(B)** *RXR*α gene expression was quantified by qRT-PCR. Data are expressed as mean ± SEM and statistical analysis employed a 2-way ANOVA with Bonferroni post-tests with ^*^p < 0.05, ^****^p < 0.0001.

Human CD4+ T cells, but not CD8+ T cells, treated with 1,25(OH)_2_D3 alone increase *SERPINA1* gene expression and AAT protein secretion (16). The requirement for TGF-β as a cofactor to promote synthesis of AAT by CD8+ T cells was investigated. Peripheral blood CD4+ and CD8+ T cells were stimulated with anti CD3/IL-2 in the presence of 10 nM or 100 nM 1,25(OH)_2_D3, with or without the addition of TGF-β1 over a broad range titration (0.02–20 ng/ml) (Figure 1). After 7 days in culture both 10 and 100 nM 1,25(OH)_2_D3 significantly increased *SERPINA1* transcription and 100 nM 1,25(OH)_2_D3 significantly upregulated AAT secretion in CD4+ T cells, but not in CD8+ T cell cultures. Addition of 2 or 20 ng/ml TGF-β1 together with the higher concentration of 1,25(OH)_2_D3 significantly increased both *SERPINA1* and AAT expression in CD8+ T cell cultures to levels similar to those observed in CD4+ T cells treated with the same concentration of 1,25(OH)_2_D3. However, the addition of TGF-β1 alone had no effect, whilst TGF-β1 with 1,25(OH)_2_D3 had no additional effect in CD4+ T cell cultures. The physiological concentration of TGF-β has been reported to be up to 4 ng/ml in human plasma (35), we therefore have selected 2 ng/ml to proceed in the study.

### TGF-β1 and 1,25(OH)_2_D3 Act Together to Upregulate *VDR* and *RXRα* Expression in CD8+ T Cells

**Figure 2:**
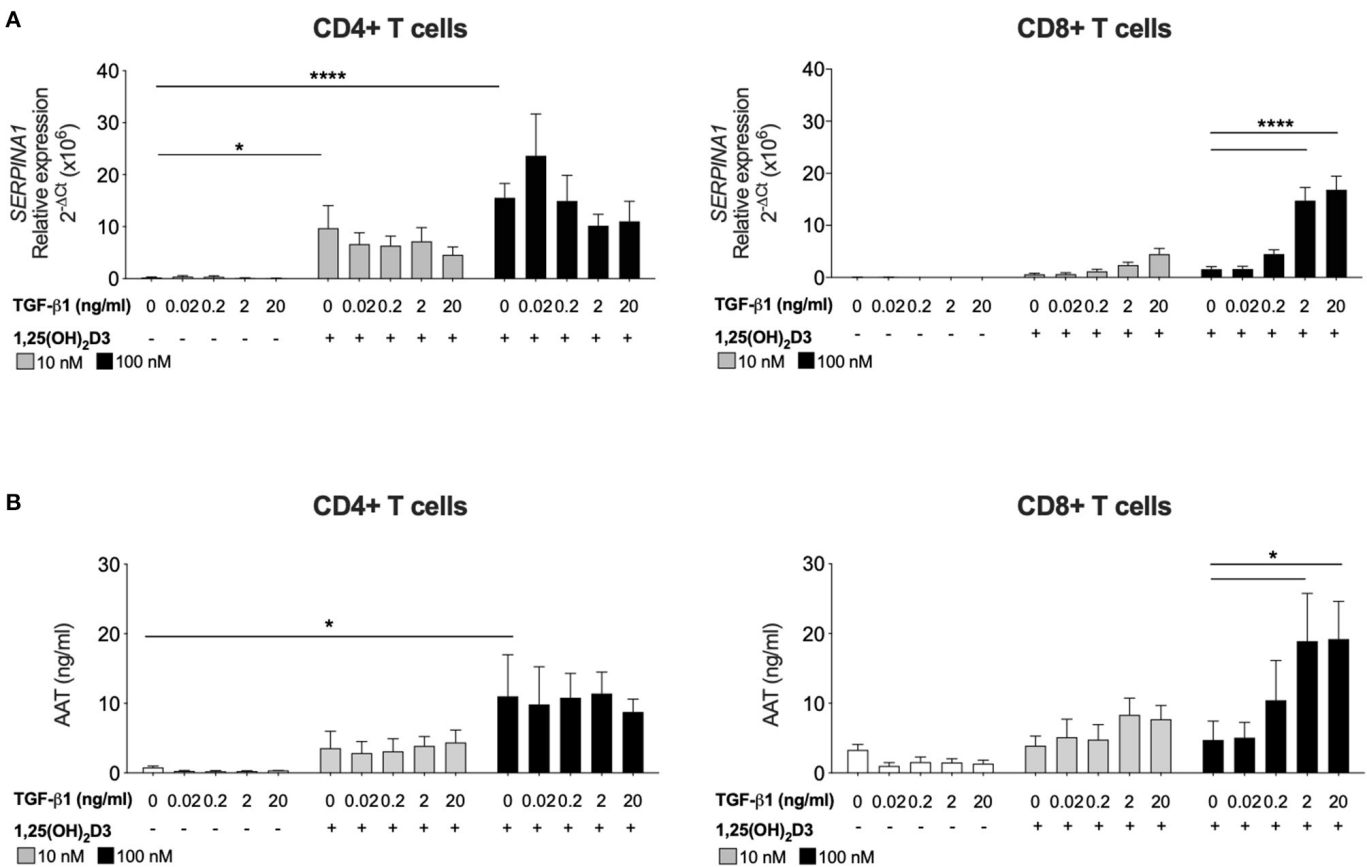
CD8+ T cells require exogenous TGF-β1 to enhance 1,25(OH)_2_D3-induced *SERPINA1*/AAT. CD4+ and CD8+ T cells (*n* = 6) were stimulated and cultured for 7 days in the absence or presence of 10 or 100 nM 1,25(OH)_2_D3, with or without TGF-β1 (0.02, 0.2, 2, 20 ng/ml). **(A)** Gene expression of *SERPINA1* was quantified by qRT-PCR. **(B)** Culture supernatants were assessed for AAT protein secreted by ELISA. Data are expressed as mean ± SEM and statistical analysis employed a 2-way ANOVA with Bonferroni post-tests with ^*^p < 0.05, ^****^p < 0.0001.

To investigate how TGF-β1 modulates the response of CD8+ T cells to 1,25(OH)_2_D3, the effect of TGF-β1 on *VDR* and *RXR*α was assessed since the VDR-RXRα complex binds 1,25(OH)_2_D3 and is essential for subsequent vitamin D-mediated effects. Addition of 1,25(OH)_2_D3 alone to cultures of CD4+ or CD8+ T cells had no effect on the gene expression of either receptor. Inclusion of TGF-β1 at higher doses (2 and 20 ng/ml) significantly enhanced 1,25(OH)_2_D3-induced (100 nM) *VDR* in both CD4+ and CD8+ T cells (Figure 2A). Expression of *RXR*α was not significantly affected in CD4+ T cells, the combination of 1,25(OH)_2_D3 and TGF-β1 significantly enhanced *RXR*α expression in CD8+ T cell cultures (Figure 2B).

### Requirement for Endogenous or Exogenous TGF-β in 1,25(OH)_2_D3-Mediated Regulation of *SERPINA1*

**Figure 3:**
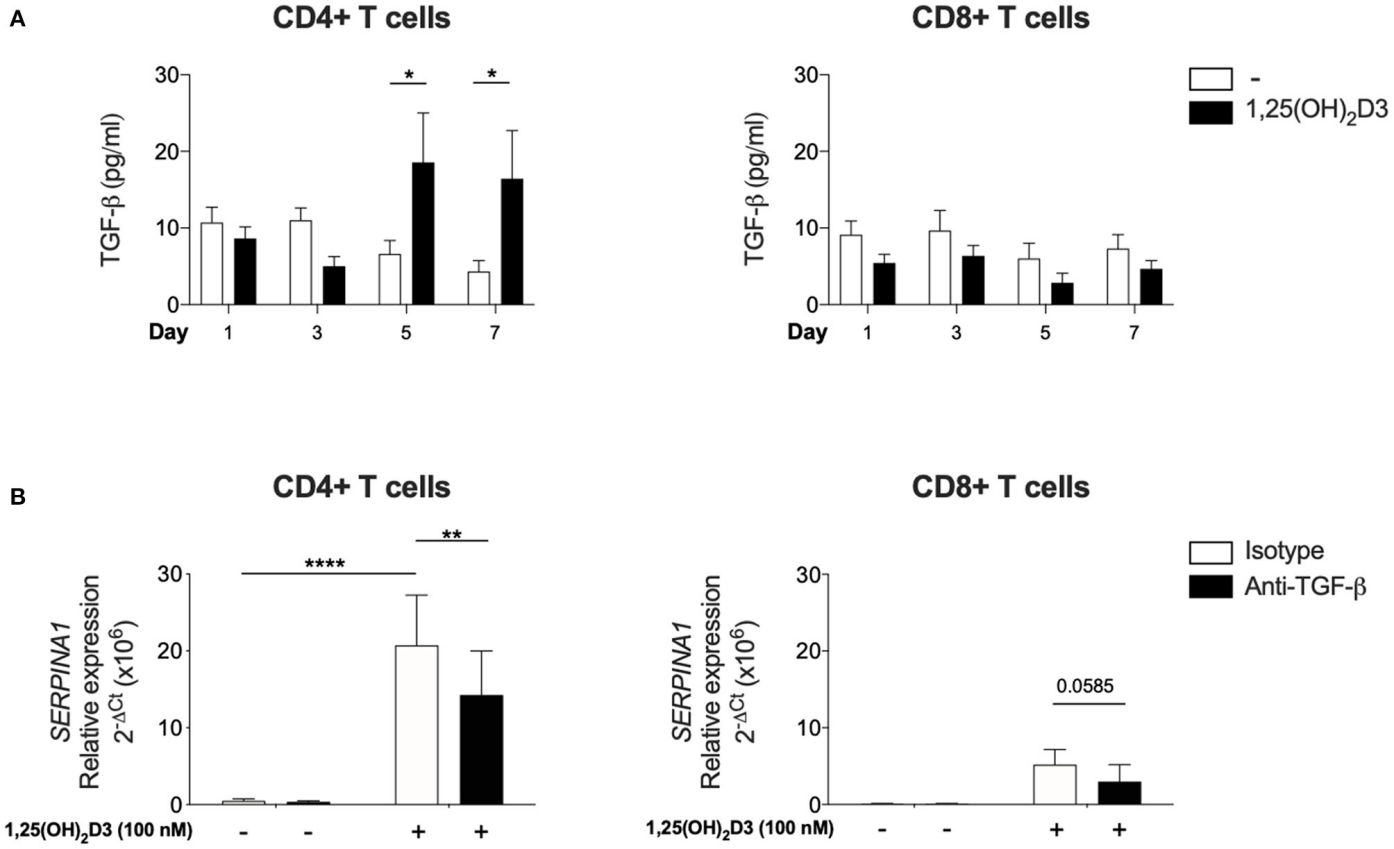
Significant upregulation of bioactive TGF-β is observed with 1,25(OH)_2_D3 treatment in CD4+ T cells and 1,25(OH)_2_D3 induction of *SERPINA1*/AAT is partially inhibited by anti-TGF-β. CD4+ and CD8+ T cells (*n* = 6) were stimulated and cultured in the absence or presence of 100 nM 1,25(OH)_2_D3. **(A)** Supernatant from indicated time points were harvested and TGF-β bioactivity was measured using transfected mink epithelial lung cells and luciferase activity (luminescence, as relative light unit corresponds to TGF-β bioactivity), and converted to pg/ml. **(B)** Cells were cultured with 100 nM 1,25(OH)_2_D3 in the presence of anti-TGF-β or an isotype control for 7 days. 1,25(OH)_2_D3-mediated induction of *SERPINA1* expression was assessed by qRT-PCR. Data are expressed as mean ± SEM and statistical analysis employed a 2-way ANOVA with Bonferroni post-tests with ^*^p < 0.05, ^**^p < 0.01, ^****^p < 0.0001.

Since CD4+ T cells did not show the same requirement for exogenous TGF-β as CD8 + T cells for *SERPINA1*/AAT induction, the issue of whether this was due to their greater endogenous production of bioactive TGF-β in comparison to CD8+ T cells was examined. Bioactive TGF-β secretion was quantified in parallel cultures of CD4+ and CD8+ T cells, using mink lung epithelial cells that were transfected with a TGF-β target gene plasminogen activator inhibitor 1 and a luciferase assay. In the absence of 1,25(OH)_2_D3, there was no difference in bioactive TGF-β levels between CD4+ and CD8+ T cells at any time point. However, bioactive TGF-β was elevated in supernatants harvested from CD4+ T cells after 5 and 7 days of culture with 100 nM 1,25(OH)_2_D3. No effect was observed in CD8+ T cells (Figure 3A).

In a complementary approach, CD4+ and CD8+ T cells were cultured in the presence of neutralising TGF-β or isotype control antibodies to assess the role of endogenous, autocrine TGF-β in controlling *SERPINA1* expression. Anti-TGF-β significantly down-regulated 1,25(OH)_2_D3-mediated *SERPINA1* expression in CD4+ T cell cultures, but in parallel CD8+ T cell cultures, 1,25(OH)_2_D3 did not significantly increase *SERPINA1*, and neutralization of TGF-β did not markedly modify this response (Figure 3B). However, modulation of AAT protein expression by neutralization of TGF-β did not achieve statistical significance (data not shown). Together, these observations indicate that the 1,25(OH)_2_D3-mediated induction of *SERPINA1* in both CD4 and CD8 T cells is at least partially TGF-β dependent. CD4+ T cells appear capable of autocrine priming with TGF-β whilst CD8+ T cells require exogenous addition of this co-factor.

### Human T Cells Respond to 1,25(OH)_2_D3 but Not Its Circulating Precursor 25(OH)D3

**Figure 4:**
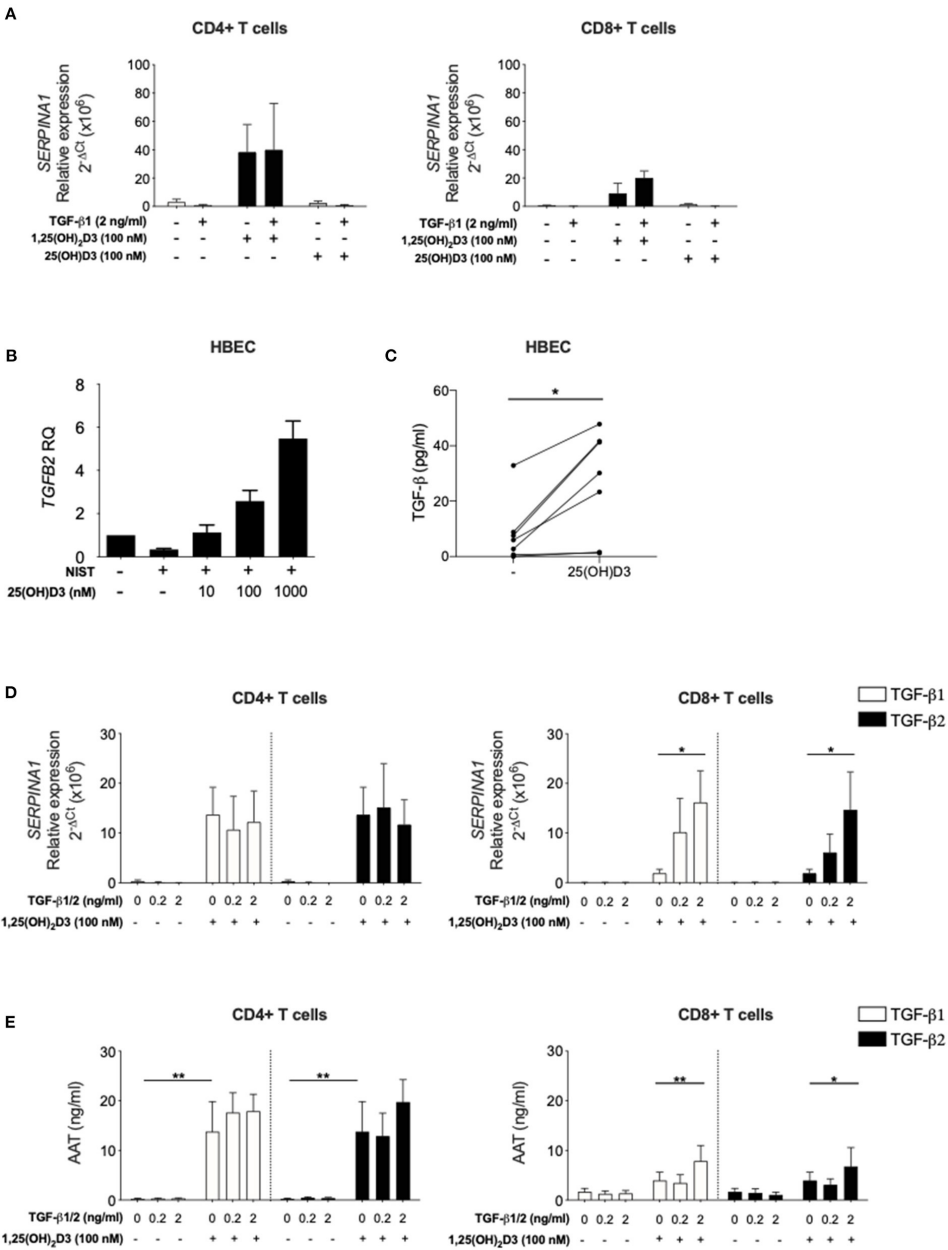
Inactive vitamin D does not induce *SERPINA1* expression in T cells, but does induce production of bioactive TGF-β in HBECs. Both TGF-β isoforms upregulate 1,25(OH)_2_D3-mediated *SERPINA1*/AAT induction in CD8+ T cells. **(A)** CD4+ and CD8+ T cells were isolated from peripheral blood of healthy donors (*n* = 4) and cultured for 7 days in the absence or presence of either 100 nM 1,25(OH)_2_D3 or 100 nM 25(OH)D, with or without 2 ng/ml TGF-β1. Cell pellets were harvested and assessed for *SERPINA1* expression. **(B)** Human bronchial epithelial cells (HBEC, *n* = 5) were analysed for *TGFB2* gene expression following 24 h stimulation with NIST and different concentrations of 25(OH)D3 (10, 100, and 1,000 nM). **(C)** HBEC (*n* = 7) were assessed for their ability to produce bioactive TGF-β in response to 48 h 1 μM 25(OH)D3. Cells were cultured for 7 days in the presence or absence of 100 nM 1,25(OH)_2_D3 with or without TGF-β1 or TGF-β2 (0.2, 2 ng/ml). **(D)** Cell pellets were assessed for *SERPINA1* gene expression (*n* = 3–6). **(E)** Culture supernatants were assessed for AAT protein secretion by ELISA (*n* = 6). Data are expressed as mean ± SEM and statistical analysis employed a 2-way ANOVA with Bonferroni post-tests with ^*^p < 0.05, ^**^p < 0.01.

The studies presented here and in our earlier publication (16) have assessed the capacity of active vitamin D (1,25(OH)_2_D3) to enhance *SERPINA1*/AAT production by T cells. Vitamin D circulates predominantly in the precursor form 25(OH)D3, which has a far longer half-life and is found at ~1,000-fold higher concentrations. CYP27B1, the Vitamin D 1-alpha-hydroxylase enzyme which converts 25(OH)D3 to 1,25(OH)_2_D3, is expressed by CD4+ T cells providing the potential for local modulation of vitamin D activity. However, the degree to which these cells are able to generate physiologically relevant concentrations of the active form remains controversial (36, 37). CD4+ and CD8+ T cells were therefore stimulated in the presence of 25(OH)D3 or 1,25(OH)_2_D3, with or without 2 ng/ml TGF-β. Upregulation of *SERPINA1* was observed in the presence of 1,25(OH)_2_D3 in the CD4+ T cell cultures, with much lower levels seen in the CD8+ T cells. Neither cell type responded to 25(OH)D3 for upregulation of *SERPINA1* (Figure 4A), suggesting that T cells may require 1,25(OH)_2_D3 generated locally by other cell types to upregulate *SERPINA1 in vivo*.

### Human Bronchial Epithelial Cells May Provide a Local Source of Active Vitamin D to Control T Cell Responses

Human bronchial epithelial cells (HBECs) are known to respond to both 25(OH)D3 and 1,25(OH)_2_D3 (32, 38) and are considered an important source of local mediators that activate immune cells in the airways (32, 39). In a recent study using transcriptional gene profiling we reported that human primary HBECs stimulated with 1,25(OH)_2_D3 increased *TGFB2* gene expression (32). Here, this observation was extended to demonstrate that primary HBECs increase *TGFB2* expression upon 25(OH)D3 stimulation in a concentration-dependent manner, when activated *in vitro* by exposure to NIST, a standard reference source of total urban particulate matter (SRM-1648a) which has been previously described (32) (Figure 4B). NIST was used as a relevant environmental stimulus of HBEC with previously observed effect on induction of inflammatory pathways (32). Increased bioactive TGF-β was evident at 48 h in HBEC culture supernatants in response to 1 μM 25(OH)D3 in culture (Figure 4C).

In order to support the concept that HBEC derived bioactive TGF-β may enable T cell responsiveness to 1,25(OH)_2_D3 for upregulation of *SERPINA1*/AAT, the capacity of TGF-β1 and TGF-β2 isoforms to enhance this response in CD8+ T cells was assessed. CD4+ and CD8+ T cells were stimulated in culture with 0 or 100 nM 1,25(OH)_2_D3 in the absence or presence of TGF-β1 or TGF-β2. Both TGF-β isoforms in the presence, but not the absence of 1,25(OH)_2_D3, significantly increased *SERPINA1* expression and AAT production in CD8+ T cell cultures. As previously observed, CD4+ T cells responded to 1,25(OH)_2_D3 with no further effect upon addition of either TGF-β preparation (Figures 4D,E).

### Correlation Between Serum Vitamin D and Bronchoalveolar Lavage AAT Concentrations in Children With Asthma

**Figure 5:**
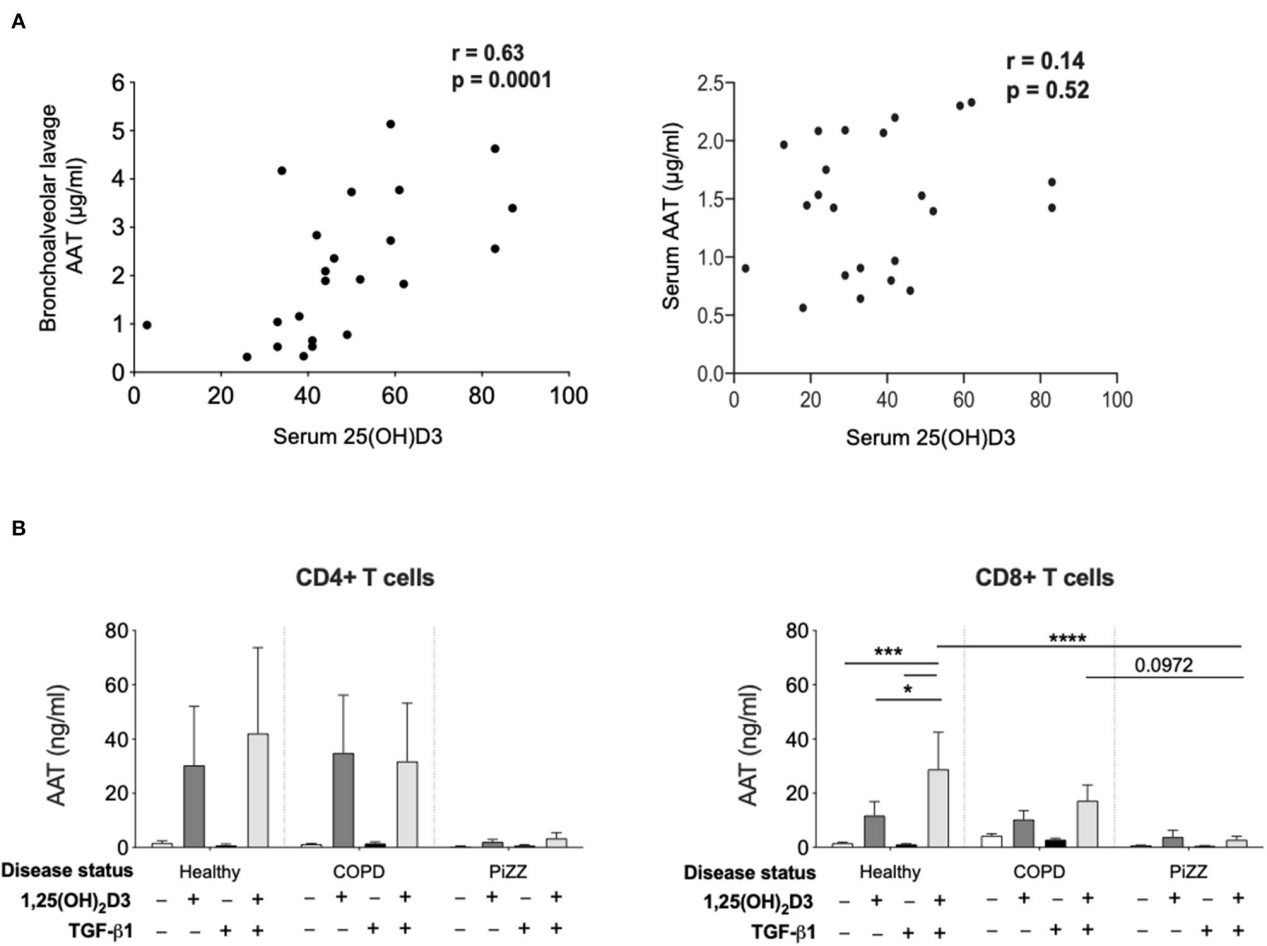
Patient studies to assess *in vivo* relevance. **(A)** Serum concentration of 25(OH)D3, as well as bronchoalveolar lavage (left) and serum (right) concentration of AAT were assessed in a paediatric severe asthma cohort and in paediatric controls undergoing bronchoscopy. Correlations were assessed using Spearman's rank correlation. **(B)** CD4+ and CD8+ T cells were isolated from the peripheral blood of healthy donors (CD4+ *n* = 6, CD8+ *n* = 12), non-AAT deficient COPD (*n* = 9) and PiZZ patients (CD4+ *n* = 6, CD8+ *n* = 20) and cultured for 7 days in the absence (–) or presence of 1,25(OH)_2_D3 (100 nM) with or without TGF-β1 (2 ng/ml) before culture supernatants were assessed for AAT protein secretion by ELISA. Data are expressed as mean ± SEM and statistical analysis employed a 2-way ANOVA with Bonferroni multiple comparison test with ^*^p < 0.05, ^***^p < 0.001, ^****^p < 0.0001.

Evidence to support the relevance of these experimental observations was explored in 2 distinct patient populations (Figure 5). At least one independent study reports a link between serum vitamin D levels and AAT levels in autoimmune disease, in this case type 2 diabetes (40). We previously described a clinically well-defined cohort of children with asthma and control children undergoing diagnostic bronchoscopies for non-asthma related investigations, in which lower vitamin D levels were associated with worse asthma control and lung function (30). Using archived serum and bronchoalveolar lavage from this cohort here we observed a significant positive correlation between serum 25(OH)D3 levels and AAT in the bronchoalveolar lavage but not the serum in this paediatric severe asthma cohort (Figure 5A). These findings suggested that circulating vitamin D availability may contribute to control AAT synthesis locally in the airways, at least in part through effects on T cells.

### Studies in Adults at Risk of COPD

Hepatocytes synthesise very high levels (1–2 g per day) of AAT, which is secreted into the circulation (41). Patients who are homozygous for the Z variant mutation (PiZZ genotype) in *SERPINA1*, produce AAT protein carrying a Glu342Lys substitution. This causes it to misfold, polymerise and aggregate within the hepatocyte such that only around 15% of normal levels of AAT protein are secreted, resulting in a similar circulating deficiency (9). These individuals represent up to 95% of the clinically diagnosed cases of AAT deficiency and are at greatly increased risk of early onset COPD. Whilst the situation in hepatocytes appears well-characterised, whether other cell types that produce lower levels of AAT will retain similar proportions of the synthesised protein as intracellular aggregates and demonstrate similarly profound secretion defects in the PiZZ context is not known. We therefore compared the levels of AAT secretion by CD4+ and CD8+ T cells treated with 1,25(OH)_2_D3 in cohorts of healthy controls, patients with COPD, and PiZZ AAT deficient individuals (Figure 5B). Clinical parameters for each group are summarized in Table 1. The AAT response was impaired in PiZZ CD4+ and CD8+ T cells (with or without TGF-β1). Simultaneous addition of 1,25(OH)_2_D3 and TGF-β1 significantly enhanced AAT secretion in healthy CD8+ T cells, and COPD CD8+ T cells showed a similar, albeit reduced trend for AAT secretion. However, PiZZ CD8+ T cells failed to secrete AAT in response to any of the treatments. Similar response profiles were observed in CD4+ cells between cohorts, but with greater variability. Overall, these observations strongly support defective secretion of AAT in PiZZ T cells, in response to stimuli that are able to increase AAT in T cells from healthy donors, consistent with the nature of the PiZZ defect in AAT secretion from hepatocytes (9). This may contribute to impaired local immunomodulation around T cells in PiZZ AAT deficiency.

## Discussion

A delicate balance between proteases and anti-proteases contributes to homeostasis in the lungs and several mediators, including vitamin D and AAT, contribute to this. This study highlights the following findings: (i) CD8+ T cells, in addition to CD4+ T cells, represent a cellular source for 1,25(OH)_2_D3-induced AAT in the airway microenvironment, (ii) TGF-β acts as a co-factor in this vitamin D controlled regulatory axis and (iii) this axis is defective in PiZZ patients with severe AAT deficiency. Based on this and our previous findings (16) we propose that T cell-derived AAT is likely to function as an intermediate for immunomodulatory roles of vitamin D in the airway (1, 5). The significant correlation between circulating levels of vitamin D with airway AAT levels in a pediatric cohort further supports a functional relationship between these two mediators *in vivo*. Our *in vitro* studies indicate this will be mediated by 1,25(OH)_2_D3 whilst implicating at least one further mediator upstream of the AAT response that we have not identified in our work to date. This work therefore provides an important starting point for further work to elucidate clear cascades of regulation and so better understand the relationship between vitamin D and AAT.

Hepatocytes are recognised as the major source of circulating AAT secreting 1–2 g/d into the circulation, however there is also good evidence for local synthesis, including in the airways (42, 43). The lower, but still significant levels of AAT secreted by T cells suggest that T cell derived AAT may act locally and have immunomodulatory functions, which have been reviewed elsewhere (44). In brief, AAT acts on structural and innate cells to increase anti-inflammatory mediators such as IL-1Ra, IL-10, to inhibit pro-inflammatory cytokine release, inhibit neutrophil chemotaxis and degranulation, and promote tolerogenic dendritic cells. In the adaptive arm, AAT increases the frequency of Foxp3 Treg cells and T cell synthesis of anti-inflammatory IL-10, whilst inhibiting pro-inflammatory mediators as well as B cell proliferation and autoantibody production. Many of these data arise from animal models where AAT is reported to control autoimmune disease, help to prevent transplant rejection and elastase-induced emphysema (45–48). Importantly, human intervention studies that build on these data are now emerging [reviewed by Song (49)]. Two recent examples report concomitant clinical and parallel immune readouts. AAT suppressed experimental graft vs. host disease (GVHD) by downmodulating inflammation, and AAT infusion was subsequently studied for treatment of steroid-resistant acute GVHD in humans. Magenau et al. reported a durable clinical response in refractory acute GVHD and that AAT administration appeared safe, with low rates of infection and with increasing ratios of T regulatory to effector T cells (50). Campos et al. (51) used double dose AAT infusion in AAT-deficient individuals to restore circulating AAT levels within the normal range, as well as to reduce serine protease activity and numerous inflammatory mediators in bronchoalveolar lavage. Additionally, findings from gene therapy studies demonstrate that low level secretion of AAT is sufficient to induce Treg responses in the local microenvironment, favouring toleration of the associated adeno-associated virus capsid (52).

Many of the immunoregulatory functions ascribed to AAT are also regulated by vitamin D. Observational studies and clinical trials indicate that vitamin D incrementally improves airway health in established chronic respiratory disease, especially in individuals who are profoundly vitamin D deficient (2, 53, 54). The capacity of vitamin D to induce antimicrobial functions, to enhance clinical and immune responses to corticosteroids, to promote epithelial barrier integrity as well as to augment multiple pathways linked to peripheral tolerance are all proposed to underpin these clinical effects (1, 5, 55). It seems probable that vitamin D and AAT demonstrate both common and independent immunoregulatory activities that contribute to airway health, however the interplay between these mediators requires further investigation.

We propose a potential role for vitamin D induced AAT synthesis by human T cells which is discussed in the context of local immune regulation in the airways. Circulating levels of 25-hydroxyvitamin D, the accepted measure of vitamin D status, correlated with AAT levels in the airways in a pediatric asthma cohort and matched non-asthmatic controls provides some support for this concept. However, it is highly unlikely that T cell-derived AAT alone accounts for this correlation. In our earlier publication we found no evidence that 1,25(OH)_2_D3 increased *SERPINA1* mRNA and/or AAT protein levels in monocytes, respiratory epithelial cells or primary hepatocytes (16). However, the capacity of vitamin D to control other pathways that regulate protease:antiprotease balance may also be relevant. This could include the previously reported downregulation by vitamin D of MMP-9 (21) which is known to inactivate AAT (56), that may further enhance the correlation between vitamin D and AAT levels in the lung.

The ChIP-Atlas database suggests there are no VDR binding sites within *SERPINA1* implying that indirect and/or non-binding effects of vitamin D may explain the association reported here. The current study does not define the mechanisms by which this may occur. However, links between circulating measures of vitamin D status and AAT levels reported here, in a study of type 2 diabetes patients (40), and suggested by a study in COVID-19 patients (57) support such an association.

Vitamin D has been shown to upregulate TGF-β levels both here and in independent studies, including in T cells and bronchial epithelial cells (32, 58). On the other hand, TGF-β has been shown to enhance VDR, with data showing that TGF-β1 increases VDR expression via SMAD3/4 in human granulosa-lutein cells (26). We speculate that the capacity of TGF-β to both independently increase AAT synthesis in hepatoma and bronchial epithelial cell lines (24, 25) as well as its capacity to enhance the response to vitamin D through increased expression of *VDR* and *RXR*α, as described here in T cells, might also contribute to the observed association between airway AAT and circulating vitamin D. Similar effects of TGF-β on VDR gene and protein expression have been reported elsewhere (26). These studies support co-operation between TGF-β1 and vitamin D to drive target gene expression. The interaction of vitamin D and TGF-β to induce an immunomodulatory response via increased local levels of AAT may contribute to the anti-inflammatory repertoire within the pleiotropic potential of TGF-β. Whether TGF-β acts to enhance the response to vitamin D by other cell types and/or other functions of interest remains to be investigated, but the latter at least is suggested by other studies in human T cells (29). As far as we are aware, whether vitamin D supplementation regulates local AAT levels in healthy or patient populations is still unknown but these data are awaited with interest.

PiZZ patients' hepatocytes synthesise normal levels of AAT mRNA and nascent polypeptide, but are unable to secrete signficant amounts of AAT into the circulation (15% of the normal circulating levels) and reduced levels are also evident in the airways (59). We previously demonstrated a loss of correlation of *SERPINA1* mRNA levels with an immunomodulatory readout, IL-10 gene expression, in PiZZ patient CD4+ T cells compared with those from controls (16). We attributed this to defective secretion of Z variant AAT protein from these cells. In hepatocytes the sequestration of Z AAT into retained polymeric species is related to the high levels of translation of the aggregation-prone variant. Our data presented here, despite the limitation of comparatively low patient numbers, confirm significantly impaired secretion of AAT is also observed in PiZZ T cells, that generally express far lower levels of AAT protein than the hepatocytes, relative to those from healthy donors. This could not be overcome by vitamin D in the presence or absence of TGF-β.

AAT deficiency such as that associated with the PiZZ mutation, is linked to an inherited increase in the risk of COPD and severe emphysema. This finding led to the paradigm that an imbalance between antiproteases and neutrophil-derived proteases contributes to the pathogenesis of emphysema (10). However, aberrant immune activation in AAT deficiency linked to lung disease also exists, with increasing support for a parallel role of anti-inflammatory properties of AAT that are lost in AAT deficiency. For example Baraldo et al. describe adaptive immune activation in T and B lymphocytes together with a marked increase in lymphoid follicles as a prominent feature in AAT deficiency (60). Similarly AAT-deficient individuals demonstrate increased circulating levels of IL-17A (61) and increased pro-inflammatory mediator profiles in the airways (44, 51).

**Figure 6:**
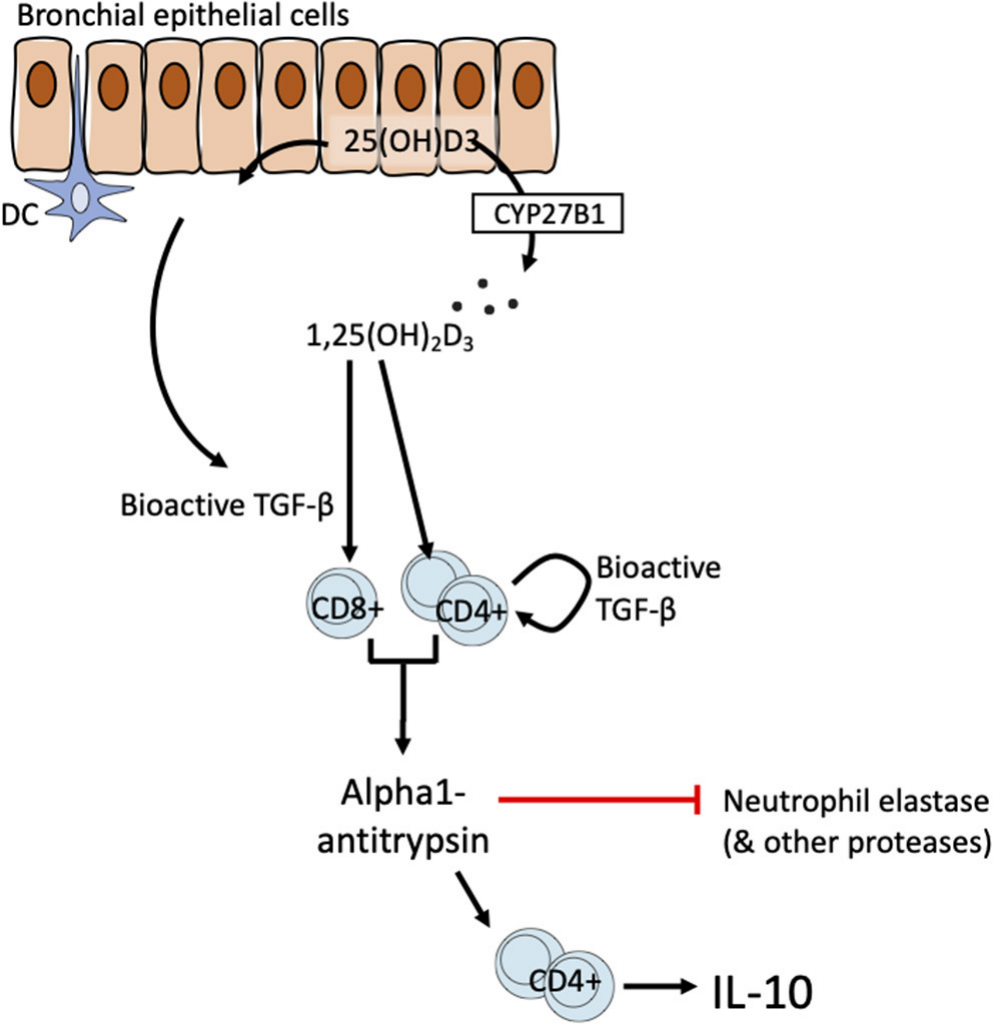
Schematic of proposed T cell-epithelial cell interaction on the vitamin D-TGF-β-AAT axis.

Bronchial epithelial cells line the airways and are amongst the first cells to be activated by exogenous stimuli such as microbes, particulate matter air pollution, allergens, and other inflammatory stimuli. They are thought to play a pivotal role in controlling immune responses in the airways through actions on dendritic cells and other cells (62). Taken together our data support the responsiveness of lung T cell and airway epithelial cells to 1,25(OH)_2_D3 as drivers of the correlation of systemic 25(OH)D3 with lung AAT levels observed *in vivo*. HBECs express CYP27B1 and therefore are able to convert precursor vitamin D to the bioactive 1,25(OH)_2_D3 (38). Our finding in HBECs further supports this by demonstrating increased bioactive TGF-β synthesis. We therefore propose a model of T cell-epithelial cell interactions in which bronchial epithelial cells may generate bioactive 1,25(OH)_2_D3 and TGF-β the latter potentially supplemented by bioactive TGF-β production in CD4+ T cells, to promote anti-inflammatory functions in T cells (Figure 6). We previously proposed a T cell-epithelial cell interaction whereby 1,25(OH)_2_D3 enhances the production of soluble ST2, the decoy receptor that antagonizes IL-33 signaling in HBECs (33). These findings together suggest a physiological supportive network between epithelial and T cells of potential benefit in the control of chronic airway disease.

For simplicity the model proposed here highlights HBECs as a likely source of TGF-β. However, a wide range of cells in the airways may also contribute including fibroblasts, smooth muscle cells and inflammatory cells. Defining the most relevant celluar source of TGF-β, and this may not reflect a single cellular source, would likely require a conditional animal knockout model to address this. The precise cellular sources of TGF-β in the microenvironment are not central to the model. TGF-β (in all isoforms) is synthesized as an inactive precursor (a latency-associated protein bound to bioactive TGF-β) that is secreted into the matrix in complex with latent TGF-β binding protein. Activation e.g., mediated by integrins, stretch, and/or proteolysis is then required for the generation of the bioactive moiety (41).

Several limitations of our findings raise questions for future research. For example, whilst we demonstrate the capacity of vitamin D to increase AAT in human T cells, the lack of an obvious vitamin D response element within *SERPINA1* suggests that intermediates in this pathway and the regulation by which this effect occurs remain to be clarified. It will also be useful to define the molecular pathways involved in local generation of the active vitamin D metabolite, 1,25(OH)_2_D3, and TGF-β required for AAT induction. Whilst a correlation between circulating 25-hydroxyvitamin D and airway AAT levels in a pediatric cohort exists, it seems unlikely that this is due solely to vitamin D induction of T cell-derived AAT and the contribution of other vitamin D regulated pathways e.g., downregulation of MMP-9, remain to be fully defined. Furthermore, these data would be complemented by evidence that vitamin D supplementation of vitamin D deficient individuals increases AAT levels *in vivo*. Finally, defining complementary, distinct and/or overlapping anti-inflammatory effects of vitamin D and AAT in patients will facilitate mechanistic and future clinical studies.

## Conclusions and Future Research Directions of This Work

In summary, our study demonstrates that vitamin D increases AAT synthesis in human T cells, via a TGF-dependent mechanism, a pathway that is impaired in individuals with a genetic mutation (PiZZ) that results in decreased levels of AAT secretion. We propose that signals from adjacent airway cells, support vitamin D-mediated induction of AAT *in situ* in human lung. Some immunoregulatory properties of AAT and vitamin D overlap such as the induction of IL-10 and increase in Foxp3-Treg frequency, leading us to propose that the role of T cell-derived AAT is most likely to be immunomodulatory and may represent an intermediate of some immunodulatory functions of vitamin D. Notably, both oral vitamin D supplementation and AAT infusion are under investigation for their capacity to inhibit inflammation in a range of human immune-mediated pathologies. For AAT these data are still limited and only now emerging (49), whilst for vitamin D these data are complicated by enormous variability in part related to variable study designs (1, 4). Nevertheless, both appear to demonstrate good safety profiles and are well-tolerated, although differences in the cost of a common oral vitamin D supplement available over the counter vs. GMP-grade manufacture of AAT, are likely to be large. Comparison of biological and immune readouts of ongoing trials may provide insight into the most pertinent, overlapping and distinct effects of the two mediators. Sole AAT augmentation therapy is currently being studied, but the potential therapeutic implication of using vitamin D supplementation as an adjuvant/combination therapy to overcome any limitations faced by sole AAT therapy may be of future interest and precedent exists for the use of vitamin D as an adjunct treatment (31, 53).

## Data Availability Statement

The original contributions presented in the study are included in the article/supplementary material, further inquiries can be directed to the corresponding authors.

## Ethics Statement

The studies involving human participants were reviewed and approved by Healthy donors (Human peripheral blood cell laboratory studies to investigate the role of immune and inflammatory pathways in respiratory disease by the local research ethics committee, REC reference: 14/LO/1699) were recruited and provided full written informed consent with full Research Ethics Committee approval. COPD patients without AAT deficiency (Chest clinic, Guy's Hospital, 14/LO/1699), and PiZZ-AAT deficient patients with or without COPD (Targeting dysfunctional mechanisms in alpha-1 antitrypsin deficiency, The Royal Free Alpha-1 clinic, REC reference: 13/LO/1085) were recruited and provided full written informed consent with full Research Ethics Committee approval. The study in a pediatric asthma cohort and control children undergoing diagnostic bronchoscopy for non-asthma related purposes was approved by the Royal Brompton and Harefield Research Ethics Committee (09/H07008/48) which has been previously described (14). Informed consent was obtained from parents and age-appropriate assent from children. Serum levels of 25-hydroxyvitamin D were measured as previously described (14). Written informed consent to participate in this study was provided by the participants' legal guardian/next of kin.

## Author Contributions

CH, BG, and Y-HC designed the study. BG recruited and screened participants. Y-HC performed functional experiments, supported by LR, SD, PP, and CC and supervised by CH and BG. SD, AG, and AB were responsible for studies in the pediatric cohort. CC, Y-HC, and CH wrote the manuscript, which all authors revised and approved. All authors contributed to the article and approved the submitted version.

## Conflict of Interest

The authors declare that the research was conducted in the absence of any commercial or financial relationships that could be construed as a potential conflict of interest.

## Publisher's Note

All claims expressed in this article are solely those of the authors and do not necessarily represent those of their affiliated organizations, or those of the publisher, the editors and the reviewers. Any product that may be evaluated in this article, or claim that may be made by its manufacturer, is not guaranteed or endorsed by the publisher.
